# The effect of spinal manipulative therapy on spinal range of motion: a systematic literature review

**DOI:** 10.1186/2045-709X-20-23

**Published:** 2012-08-06

**Authors:** Mario Millan, Charlotte Leboeuf-Yde, Brian Budgell, Martin Descarreaux, Michel-Ange Amorim

**Affiliations:** 1 EA 4532 CIAMS, UFR STAPS, Univ Paris-Sud, Orsay, France; 2 The Research Department, The Spine Centre of Southern Denmark, Hospital Lillebælt, Odense, Denmark; 3 Institut Franco-Européen de Chiropratique, Paris, France; 4 Institute of Regional Health Services Research, Faculty of Health Sciences, University of Southern Denmark, Odense, Denmark; 5 Canadian Memorial Chiropractic College, Toronto, ON, Canada; 6 Département de Chiropratique, Université du Québec à Trois-Rivières, Trois-Rivières, QC, Canada; 7 Institut Universitaire de France, Paris, France

## Abstract

**Background:**

Spinal manipulative therapy (SMT) has been shown to have an effect on spine-related pain, both clinically and in experimentally induced pain. However, it is unclear if it has an immediate noticeable biomechanical effect on spinal motion that can be measured in terms of an increased range of motion (ROM).

**Objective:**

To assess the quality of the literature and to determine whether or not SMT is associated with an immediate increase in ROM.

**Design:**

A systematic critical literature review.

**Method:**

Systematic searches were performed in Pubmed, the Cochrane Library and EMBASE using terms relating to manipulation, movement and the spine. Selection of articles was made according to specific criteria by two independent reviewers. Two checklists were created based on the needs of the present review. Articles were independently reviewed by two reviewers. Articles were given quality scores and the data synthesized for each region treated in the literature. Findings were summarized in tables and reported in a narrative fashion.

**Results:**

Fifteen articles were retained reporting on experiments on the neck, lumbar spine, hip and jaw. The mean quality score was 71/100 (ranges 33/100 - 92/100). A positive effect of SMT was reported in both studies where mouth opening was assessed after cervical manipulation. In five of the nine studies on cervical ROM a positive effect was reported, whereas the remaining four studies did not show improvement. None of the three studies of the lumbar spine showed an effect of SMT on lumbar ROMs and one study of sacroiliac manipulation reported no effect on the ROM of the hip joint.

In relation to the quality score, the seven highest ranked studies, showed significant positive effects of SMT on ROM. Continuing down the list, the other studies reported no significant differences in the outcomes between groups.

**Conclusion:**

SMT seems sometimes to have a small effect on ROM, at least in the cervical spine. Further research should concentrate on areas of the spine that have the potential of actually improving to such a degree that a change can be easily uncovered.

## Introduction

It is well known that back pain is highly prevalent in the general population, with serious economic consequences both on an individual and societal level. One of the treatments frequently used for back pain is spinal manipulative therapy (SMT). Although it has been shown that SMT is a reasonable treatment option for some people and some conditions [1], the mechanisms by which SMT achieves its effects remain unclear.

A manipulation is said to occur when a joint is brought to the extreme of its passive range and thereafter submitted to a rapid but shallow thrust, a so-called high velocity low amplitude (HVLA) manipulation [2]. The manipulation has, indeed, been shown to be a mechanical event as it causes slight momentary deformations of the spine and surrounding soft tissues [3-5]. It has also been confirmed that SMT carries the vertebrae beyond their normal physiological range of motion (ROM) without exceeding the boundaries of anatomic integrity [5,6]. Vertebral rotations and facet joint capsule strain magnitudes during SMT have been shown to be within the ranges that occur during physiological movements [6]. This maneuver is often accompanied by a crack [7].

SMT is usually differentiated from mobilization, which is described as more of a slow, long-lasting stretch of an articulation at the extreme of its passive range [8,9] and usually without causing a crack. Many clinicians make a strict distinction between SMT and mobilization [10] but some clinical studies have shown the effects to be similar for the two [11], indicating that such a distinction may not be relevant, at least not from a clinical point of view. Furthermore, the literature reports that, in clinical practice, the external forces applied during HVLA treatments vary considerably depending upon the treatment site, clinician and the technique used [7,12].

To better understand the true role of SMT in health care, more information is needed regarding the effects of SMT on the articular and peri-articular structures, and joint function; i.e. movement. Theoretically, SMT could have an effect on a number of structures and tissues within and surrounding the spinal articulation. These structures include muscles, aponeuroses, peri-articular receptors, tendons, discs, and ligaments, which all, singly or in combination, would have the capacity to cause dysfunction and pain as they are all potential starting points of proprioceptive and nociceptive pathways. For SMT to have a positive effect on vertebral movement, it must modulate the function of some or all of these structures/tissues. SMT has, in fact, been shown to modify spinal reflex excitability [13], paraspinal cutaneous temperature [14], visceral activity (e.g. cardiovascular function) [15], and electromyographic activity of paraspinal muscles [16,17]. It has also been hypothesized to alter motoneuron excitability [18], increase muscle strength [19,20], alter sensorimotor integration [21], and affect pain regulation in the dorsal horn of the spinal cord [22] and the periaqueductal grey area [23-25]. However, it is unclear as to what this brings in terms of a biomechanical effect, such as an increased range of movement (ROM). It has already been shown that SMT has a pain reducing effect [26,27]. It seems logical that if pain is reduced after SMT, ROM might increase, independently of any effect that it may have on pain. However, ROM might well increase after SMT, regardless of whether the treated segment was painful or not. Therefore, it is important to consider the effect that SMT may have on ROM, specifically. Because the literature is unclear on this topic, this systematic critical literature review was performed to assess the quality of the available literature and to determine whether or not SMT is associated with an immediate increase in ROM.

## Methods

Design: systematic critical literature review.

Searches were conducted in PubMed, the Cochrane library and EMBASE without any date limitations and using the following combinations of search terms:

 •“spinal” + “manipulation” + “range” + “motion”

 •“chiropractic” + “manipulation” + “range” + “motion”

 •“spinal” + “manipulation” + “stiffness”

 •“spinal” + “mobilization” + “range” + “motion”

 •“spinal” + “mobilization” + “stiffness”

Thereafter, two of the authors (MM and CLY) independently applied specific inclusion criteria to select the potentially relevant articles from the titles, abstracts, and keywords of the references retrieved in the literature search. These inclusion criteria were:

 •Languages: English, French, Spanish, Danish, Swedish and Norwegian.

 •Spinal Manipulative Therapy (SMT), anywhere in the spine.

 •Humans or animals.

 •Experimental studies with at least one control group. Thus, studies without a random allocation procedure were accepted.

 •ROM had to be one of the outcome variables.

 •Immediate effect was reported. (This review did not examine long term effects at, for example, weeks or months following SMT, due to the risk of contamination from factors other than the manipulative procedure.)

The following studies were excluded:

 •Studies of subjects with specific pathologies, such as inflammatory diseases, tumors or severe spinal degeneration, because these might influence the spinal structures in such a way as to limit ROM permanently and therefore confuse the results.

 •Studies of combined/concomitant therapies (drugs, physiotherapy), as it would not be possible to know if the results were obtained from SMT or the other therapies.

 •Literature reviews

### Data extraction

Two authors (MM and CLY) extracted data from each article, independently of each other, into two check-lists, one descriptive and one qualitative. Data were later compared in order to minimize reading errors. The other authors were designated as judges in case of disagreement between the first two reviewers.

### Descriptive check-list

The descriptive checklist for the selected articles (see Table 1) contained the following items:

 •ID n°; author and year of publication;

 •Study design;

 •Type of study sample;

 •Number of study subjects;

 •Numbers of males and females;

 •Age range and mean/median age of final study sample;

 •Type and area of symptoms (if any);

 •Duration of symptoms (if any);

 •Description of the SMT/mobilization group and the type(s) of control group (sham treatment, another treatment or no treatment at all).

 •Number of experiments performed on each study subject;

 •Time when ROM was measured (immediately after the SMT or later);

 •Whether SMT was performed at the level of a spinal complaint;

 •Type of measurement (distance or angle) ;

 •Method of measurement;

 •Approval from ethics committee (yes/no)

**Table 1:** Descriptive check-list of selected articles in the literature review of the action of SMT on ROM

**Author year and references**	**Design**	**Type of study sample**	**n° of subjects**	**n° males**	**n° females**	**Ages**	**Type and area of symptoms (if any)**	**Duration of symptoms**	**Treatment and control activity (sham, other or nothing)**	**N° experiments**	**When was ROM measured?**	**Was SMT performed at level of problem?**	**What was measured?**	**How was ROM measured?**	**Approval from ethics committee?**
Cassidy 1992 [36]	RCT	Patients	100	?	?	34.5 SD13.0	Unilateral neck pain with radiation to trapezius	<1week n=16 1w - 6m=34 >6 months n=50	- SMT (cervical, clinically determined) (n=52) - Mobilization (cervical) (n=48)	1	Before and 5’ after	Yes	Cervical ROM, 3 planes	Goniometer 3 D	Yes
Pollard 1997 [48]	RCT	Chiropractic students	34	?	?	21-33	None	N/A	- Sacroiliac manipulation (n=18) - Sham=Mastoid pressure (n=16)	1	Before and 30” after	N/A	Straight leg raise on SMT side	Digital goniometer	Yes
Goodsell 2000 [45]	Cross-over	Patients	26	13	13	(16–69) 39.5	Low back pain	0.5 – 60 months	- Clinically determined lumbar mobilization - Nothing (lying down)	2	Before and after	Yes	Lumbar motion (extension/flexion)	Fingertips-to-floor Inclinometer	?
Whittingham 2001 [40]	Cross-over	Volunteers obtained through adverts	105	42	63	Group=39.4 Group2=41.9	Cervicogenic headache occipital region	> 6 months	- SMT upper cervical - Sham (deactivated instrument)	3	Before and after weeks 3, 6, 9	Yes	Lateral flexion, rotation	Strap-on head goniometer	Yes
Martinez Segura 2006 [37]	RCT	Patients referred to physiotherapy to osteopathic clinic	70	25	45	(20–55) 37	Neck pain	>1Month	- SMT (C3-C5) - Sham neck mobilization	1	Before and 3’ after	Yes	Cervical ROM, 3 planes	Goniometer	Yes
Konstantinou 2007 [46]	Cross-over	Patients referred for physiotherapy suitable for SMT	26	15	11	>18 38.3 SD11.7	Low back pain +/− Leg pain	>3 months	- PA mobilization - Nothing	2	Before and 5’ after	Yes	Lumbar flexion/extension	Double inclinometer	Yes
Tuttle 2008 [41]	Cross-over	Volunteer staff and students from university	20	10	10	(19–55) 31	Neck pain that limited movement	>2 weeks	- PA cervical mobilization at symptom level - Placebo (PA mobilization but asymptomatic side) - Nothing (lying down) - general MT but no high velocity thrust	4	Before and after	Yes	Cervical ROM 3 planes	Post/ant assessment device and head sensor	Yes
Krauss 2008 [42]	RCT	Patients referred for physical therapy	32	6	26	(19–50) 34.2	Neck pain	?	- Thoracic SMT - Nothing	2	Before and after	No (thoracic adjustment)	Active ROM - Left/right rotation	Inclinometer compass system	Yes
Kanlayanaphotporn 2009 [43]	RCT	Patients	60	24	36	Goup1 39.7 Goup2 44.8	Neck pain	Mean Goup1 804d Goup2 999d	-Clinically determined mobilization - Random mobilization	1	Before and 5’ after	Yes	Cervical ROM, 3 planes	Magnetic neck brace + 3 inclinometers	Yes
Mansilla-Ferragut 2009 [34]	RCT	Volunteers obtained through advert	37	-	37	(21–50) 35+/−8	Neck pain < 40 mm mouth opening	>6 months	- SMT atlanto-occipital - Sham (cervical manual contact)	1	Before and after	Yes (atlanto-occipital)	Mouth opening	Universal caliper	Yes
Mc Clatchie 2009 [38]	Cross-over	Orthopedic patients	21	7	14	49.8 (+/−9.8)	Shoulder pain No neck pain last year	>6 weeks	- Mobilization (C5- C7) - Sham (same mobilization position but without external force)	2	Before and after	Yes	Cervical ROM, 3 planes	Goniometer	Yes
Passemore 2010 [39]	RCT	Volunteer chiropractic students with C1-C2 fixation	15	?	?	21-42	N/A	N/A	- SMT(C1-C2) - Nothing (wait 5’)	1	Before and 5’ after	N/A	Cervical ROM, 3 planes	Goniometer	Yes
Kanlayana-photporn 2010 [44]	RCT	Patients	60	18	42	42.2 (23–68)	Neck pain	Mean Group1 1575d Group2 1811d	- Post/ant (PA) cervical mobilization - random mobilization (PA, right or left)	1	Before and after	Yes	Cervical ROM 3 planes	Magnetic neck brace + 3 inclinometers	Yes
Oliveira Campello 2010 [35]	RCT	Volunteer students	122	31	91	18-30	N/A	N/A	- SMT atlanto-occipital - Soft occipital tissue treatment - Nothing	1	Before and 2’ after	N/A	Mouth opening	Universal caliper	Yes
Stamospapastomous 2011 [47]	Cross-over study	Volunteer students	32	16	16	25.5 SD4.5	N/A	N/A	- SMT(lumbar) - Mobilization (lumbar)	2	Before and after	N/A	Bending stiffness and ROM lumbar extension/flexion	Electromagnetic tracking device	Yes

### Quality checklist

A number of issues related to quality of study were identified by consensus among the authors and used to develop a quality checklist (see Table 2). We designed this checklist based on concepts presented in the PRISMA statement [28], the CONSORT statement [29] and Cochrane guidelines [30] bearing in mind that there can be no general recipe for such work, as review procedures have to be topic specific. The risk of bias was assessed following the criteria suggested by the method guidelines for systematic reviews of trials of treatments for neck and back pain by Furlan et al. [30]. Additional items were mainly adapted from PRISMA [28]. Thus, the items selected for our review were:

**Table 2:** Quality check-list of articles reviewed

**Author and references**	**Random allocation**		Study subjects naïve*/disinterested		**Was there a credible sham treatment?**		**Was SMT well described?**		**Was SMT performed by experienced person?**		**Was the ROM assessor blinded?**		**Were pre/post treatment ROM-test performed by the same person?**		**Was ROM assessed by a valid method or own inter/intra validation?**		**Were losses reported?**		**Were results reported and estimates shown in tables/graphs?**		**Were results tested for statistical significance?**		**Physical conditions controlled for all treatment groups**		**If study samples contained people older than 45, was the age distribution shown in treatment groups to be similar?**		**Total score**	**Score /100**
Oliveira Campello [35]	Yes	1	Yes	1	Yes	1	Yes	1	Yes	1	Yes	1	Yes	1	Yes	1	Yes	1	Yes	1	Yes	1	?	0	N/A	-	11 /12	**92**
Kanlayanaphotporn [43]	Yes	1	No	0	Yes	1	Yes	1	Yes	1	Yes	1	Yes	1	Yes	1	Yes	1	Yes	1	Yes	1	?	0	No, but compared base line variable for both	-	10 /12	**83**
Konstantinou [46]	Yes	1	Yes	1	No	0	Yes	1	Yes	1	Yes	1	Yes	1	Yes	1	Yes	1	Yes	1	Yes	1	?	0	Upper level age is known, but N/A because cross-over	-	10 /12	**83**
Whittingham [40]	Yes	1	Yes	1	Yes	1	Yes	1	?	0	Yes	1	Yes	1	Yes	1	Yes	1	Yes	1	Yes	1	?	0	Age range are known, but N/A because cross-over	-	10 /12	**83**
Stamospapastomous [47]	Yes	1	Yes	1	Yes	1	Yes	1	Yes	1	No	0	Yes	1	Yes	1	Yes	1	Yes	1	Yes	1	?	0	N/A	-	10 /12	**83**
Krauss [42]	Yes	1	Yes	1	No	0	Yes	1	Yes	1	Yes	1	Yes	1	Yes	1	Yes	1	Yes	1	Yes	1	?	0	No (19 – 50)	0	10 /13	**77**
Martinez Segura [37]	Yes	1	Yes	1	Yes	1	Yes	1	Yes	1	Yes	1	Yes	1	Yes	1	No	0	Yes	1	Yes	1	?	0	No (20 – 55)	0	10 /13	**77**
Goodsell [45]	Yes	1	Yes	1	No	0	Yes	1	Yes	1	Yes	1	Yes	1	Yes	1	No	0	Yes	1	Yes	1	?	0	Yes	1	9 /13	**69**
Mc Clatchie [38]	Yes	1	Yes	1	Yes	1	Yes	1	?	0	Yes	1	Yes	1	Yes	1	No	0	Yes	1	Yes	1	?	0	No (>16)	0	9 /13	**69**
Mansilla-Ferragut [34]	Yes	1	?	0	Yes	1	Yes	1	Yes	1	Yes	1	Yes	1	Yes	1	No	0	Yes	1	Yes	1	?	0	No (21–50)	0	9 /13	**69**
Cassidy [36]	Yes	1	Yes	1	Yes	1	Yes	1	Yes	1	Yes	1	?	0	Yes	1	No	0	Yes	1	Yes	1	?	0	Age not known	0	9 /13	**69**
Tuttle [41]	Yes	1	?	0	Yes	1	No	0	Yes	1	Yes	1	Yes	1	Yes	1	No	0	Yes	1	Yes	1	?	0	Age range known, but N/A because cross-over	-	8 /12	**67**
Kanlayanaphotporn [44]	Yes	1	No	0	Yes	1	Yes	1	Yes	1	Yes	1	No	0	Yes	1	No	0	Yes	1	Yes	1	?	0	No, but compared base line variable for both	-	8 /12	**67**
Passmore [39]	Yes	1	No	0	No	0	Yes	1	Yes	1	Yes	1	?	0	?	0	No	0	Yes	1	Yes	1	?	0	N/A	-	6 /12	**50**
Pollard [48]	Yes	1	No	0	No	0	Yes	1	?	0	?	0	?	0	?	0	No	0	Yes	1	Yes	1	?	0	N/A	-	4 /12	**33**

#### Study population

 •1. Ideally, study subjects should be naïve or at least disinterested, meaning that they should not have any preconceived ideas about SMT that could affect their reactions to the experiment. This would exclude, for example, studies of chiropractic students. Patients referred for physiotherapy treatment and thereafter included in a study were considered suitable for inclusion because they did not actively select participation in the study.

 •2. If the study samples contained people older than 45, the age distribution should be similar among the treatment groups, as an uneven number of older people could influence the degree of spinal degeneration and so response to treatment as degeneration has been shown to modify the effect of SMT [31,32].

#### Procedure

 •3. The study subjects should have been randomly allocated to study groups.

 •4. Sham treatments, if any, should be credible, to reduce the risk of negative expectations from study subjects.

 •5. Physical conditions, such as room temperature, time of day, time of warm-ups, should be similar for all treatment groups, and also constant before/after SMT because such conditions might have an effect on spinal movement [33].

 •6. The manipulative maneuver should be well described to make it possible to ascertain whether it was actually a manipulation, but also to make it possible to reproduce the study.

 •7. The manipulative maneuver should be performed by an experienced person, to ensure that it was correctly carried out.

 •8. The person assessing the ROM should be blinded to treatment group to ensure absence of expectation bias.

 •9. The pre/post treatment ROM-test should be performed by the same person to optimize the reliability of the test procedure.

 •10. The ROM should be assessed by a method previously shown to be highly reliable or the study should contain a satisfactory report of own inter/intra reliability evaluation.

#### Data reporting

 •11. Losses and exclusions should be reported to minimize the risk of selective removal of data.

 •12. Estimates should be reported as exact values or shown in tables/graphs and not only as significance levels.

 •13. If there were any differences between groups, these should be tested for statistical significance.

### Classifying articles by their quality

For the selected articles, all descriptive and quality items were checked independently by the first two reviewers and a summary quality score was assigned. One point was awarded for each fulfilled quality item.

If the quality item was not satisfied (“no”) or if the article did not mention this condition (”?”), no point was given. However, if the item was not applicable (“N/A”), this was not counted in the denominator. Thus the maximum total score could be different from one article to the next. Finally, the total score was normalized to a 100 point scale in order to compare quality across studies.

Although a summary quality score was calculated, no cutoff-point was defined for acceptable or unacceptable level of quality. This allowed us (and the readers) to use the quality scores and the information on each individual quality item as a guide to determine whether articles would be considered more or less credible. In other words, the quality assessment was meant to be informative rather than proscriptive. Because we decided not to use a scoring system to establish levels of quality, we have not defined any discrete levels of evidence, e.g. high, moderate or low.

### Data synthesis

Four data tables were constructed according to the area of the spine where treatment was provided and ROM assessed: 1. cervical treatment and ROM, 2. lumbar treatment and ROM, 3. cervical treatment and range of mouth opening, and 4. sacroiliac treatment and hip ROM. The tables were scrutinized, one by one, to obtain answers to our research question. Results were thereafter interpreted and reported in a narrative fashion.

## Results

### Study selection

**Figure 1:**
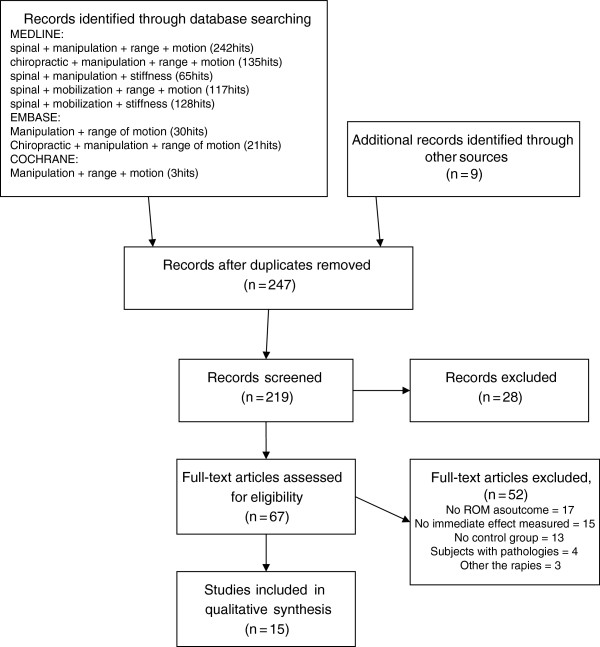
**Flowchart describing the process for a systematic critical review of the literature on the effect of SMT on spinal range of motion (from The PRISMA Statement [**[56]**])**

Figure 1 shows a flow diagram of the study selection process. A total of 242 articles were identified from the systematic search of MEDLINE, 51 from EMBASE, and 3 from the Cochrane library. Nine other articles were added from an additional hand search of reference lists. After duplicates were removed, 247 articles remained to be assessed for inclusion. Of these, 67 were retrieved in full and examined against our inclusion criteria. In all, 15 studies fulfilled all inclusion criteria and are reviewed herein (see Figure 1).

### Description of data

Table 1 provides a full description of the key characteristics of each study. Articles have been listed chronologically based on the date of publication.

Of the 15 selected studies, two measured the effects of SMT on mouth opening, nine studied effects about the three cervical axes of movement, one measured only the range of cervical rotation, three studied flexion/extension in the lumbar spine, and one investigated the ROM of the hip. There were nine randomized controlled trials and six cross-over studies. All of them measured the ROM before and immediately after treatment. No papers on the thoracic spine satisfied the inclusion criteria.

ROM was measured differently depending upon the spinal region under study. Mouth opening ( [34] and [35]) was measured with a universal caliper. Cervical ROM was assessed with a three- dimensional goniometer ( [36], [37], [38] and [39]), a strap-on head goniometer ( [40]), a postero-anterior assessment device with a head sensor ( [41]), an inclinometer with a compass system ( [42]), and a magnetic neck brace with three separate inclinometers ( [43] and [44]). Lumbar ROMs were measured with a fingertip-to-floor inclinometer [45], a double inclinometer [46] and an electromagnetic tracking device [47]. Hip ROM was measured with a digital goniometer ( [48]).

Some researchers (n=11) performed their experiments on people with symptoms, either patients (n=8) or volunteers with symptoms recruited by advertisements (n=3), whereas the remaining four studies dealt with healthy students.

Eight authors performed only one experiment, five reported two experiments, and two studies reported on three and four experiments, respectively.

Only one article did not report whether approval had been received from an ethics committee.

### Quality

Table 2 presents a description of the quality items. The quality scores ranged from 33/100 to 92/100, with a mean score of 71.4 (SD 10.4) and a median of 69. Articles are listed in the order of decreasing quality score. There were no disagreements between the reviewers in relation to the quality items.

All articles fulfilled the following four quality criteria: “Random allocation”, “SMT well described”, “Estimates reported and shown in tables/graphs” and “Results tested for statistical significance”. The item “Age distribution similar in studies with study sample consisting of people older than 45” was applicable to six studies ( [36], [45], [37], [42], [34], and [38]) of which only [45] fulfilled the criterion.

Nine articles dealt with “disinterested/naïve study samples”. Ten studies included what we considered a credible sham treatment in comparison to the spinal manipulation. However, some studies investigated differences in outcome in relation to different types of manipulation (such as manipulating in different planes), i.e. not comparing manipulation to a sham treatment making this issue superfluous. Eleven authors specified that SMT was performed by an experienced person (all except [36,39,44,48]), and twelve assured that pre/post assessment was made by the same person (all except [36,39,48]).

Outcome assessments were blinded in thirteen studies, and thirteen assessed ROMs with a validated method. In two of the studies, ( [48] and [39]), the validity of the assessment method was not described.

In none of the studies were the experimental conditions stated to have been equal for treatment and control groups or before and after the manipulation. Only six studies reported if there were any exclusions or losses during the study.

### Outcomes reported

The results for mouth opening, cervical ROM, lumbar ROM and hip ROM are shown in Tables 3, 4, 5 and 6 and are described below.

**Table 3:** Effects of SMT on mouth opening

**Author**	**Quality score /100**	**Treatment and control activity (sham, other or nothing)**	**Preintervention in mm**	**Postintervention in mm**	**Change of score in mm**	**Summarized results given by authors**	**Were the differences in outcome tested between groups and were they significant?**
Oliveira Campello [35]	**92**	- SMT atlanto-occipital (n=41) - Soft occipital tissue treatment (n=41) - Nothing (n=40)	46.4 ± 6.8 (44.4, 48.4) 47.2 ± 6.2 (45.2, 49.3) 46.8 ± 6.8 (44.8, 48.9)	47.9 ± 6.8 (45.9, 49.9) 47.7 ± 6.1 (45.6, 49.7) 46.8 ± 6.7 (44.8, 48.9)	**1.5** ± 1.5 (1.0, 1.9) **0.5** ± 1.7 (0.0, 1.0) **0.0** ± 1.1 (−0.4, 0.3)	SMT increases maximum active mouth opening, but need of further studies to elucidate the clinical relevance of that.	Yes and yes
Mansilla-Ferragut [34]	**69**	- SMT atlanto-occipital (n=18 ) - Sham (cervical manual contact) (n=19 )	35.4 (95% CI, 33.3-37.4) 36.2 (95% CI, 34.3- 38.2)	38.8 (95% CI, 36.6-41.1) 35.9 (95% CI, 33.7-38.0)	**3.5** (95% CI, 2.4, 4.6) −**0.3** (95% CI, -0.4, 1.2)	SMT increases maximum active mouth opening.	Yes and yes

**Table 4:** Effects of SMT on cervical range of motion

**Author**	**Quality score /100**	**Treatment and control activity (sham, other or nothing)**	**Δ ROM pre/post treatment in degrees: flexion**	**Δ ROM pre/post treatment in degrees: extension**	**Δ ROM pre/post treatment in degrees: right lateral flexion**	**Δ ROM pre/post treatment in degrees: left lateral flexion**	**Δ ROM pre/post treatment in degrees: right rotation**	**Δ ROM pre/post treatment in degrees: left rotation**	**Summarized results given by authors**	**Comments**	**Were the differences in outcome tested between groups and were they significant?**
Kanlayanaphotporn [43]	**83**	- Clinically determined mobilization - Random mobilization	1.9 ± 4.1 −0.7 ± 4.5	1.8 ± 6.3 0.8 ± 4.6	- 0.3 ± 6.3 1.2 ± 4.9	0.8 ± 4.8 1.3 ± 4.1	0.5 ± 5.5 0.6 ± 6.9	1.1 ± 4.2 0.8 ± 6.0	The preferred mobilization group showed an increased flexion/extension compared to random mobilization group.		Yes and yes
Whittingham [40]	**83**	- SMT upper cervical - Sham (deactivated instrument)	-	-	Gr. 1=39° ± 1.1 Gr. 2=38° ± 1.4	Gr. 1=38° ± 1.3 Gr. 2=36° ± 1.2	Gr. 1=56° ± 1.4 Gr. 2=57° ± 1.5	Gr. 1=54° ± 1.6 Gr. 2=54° ± 1.6	SMT increase significantly ROM	Those are given baseline values. ROM values immediately after treatment. Effect is observed on weeks 3, 6, 9 and 12.	Yes and yes
Krauss [42]	**77**	- Thoracic SMT - Nothing	-	-	-	-	8.23 (SD=7.41) −0.1 (SD=2.33)	7.09 (SD=5.83) −0.6 (SD=3.66)	Cervical rotation ROM is improved following SMT		Yes and yes
Martinez Segura [37]	**77**	- SMT (C3-C5) - Sham neck mobilization	7 (Cohen’s d=5) 1.5 (Cohen’s d=2.5)	8 (Cohen’s d=7) 1.4 (Cohen’s d=3.3)	5 (Cohen’s d=4) 0.8 (Cohen’s d=1.6)	5 (Cohen’s d=4) 0.8 (Cohen’s d=1.5)	10 (Cohen’s d=5) 0.4 (Cohen’s d=1.5)	9 (Cohen’s d=5) 0.3 (Cohen’s d=0.8)	SMT was more effective than control mobilization on ROM. Large effect sizes.		Yes and yes
Mc Clatchie [38]	**69**	- Mobilization (cervical) - Sham (same mobilization position but without external force)	−1.2 ± 6.5 −1.4 ± 5.3	0.8 ± 5.5 −0.5 ± 5.5	−0.7 ± 5.2 −0.1 ± 5.3	−0.4 ± 4.1 0.3 ± 4.4	1.1 ± 4.4 −0.4 ± 5.9	1.3 ± 6.6 - 0.3 ± 4.9	No significant difference		Not tested
Cassidy [36]	**69**	- SMT(cervical) (n=52) - Mobilization (cervical) (n=48)	5.1 (SD 8.3) 3.9 (SD 9.4)	3.1 (SD 7.8) 1.3 (SD 7.5)	3.4 (SD 7.5) 2.0 (SD 5.2)	4.3 (SD 7.0) 3.0 (SD 4.7)	5.0 (SD 9.0) 4.2 (SD 9.0)	3.6 (SD 7.0) 2.4 (SD 6.4)	Both treatments increase ROM to similar degree.		Yes and no
Kanlayanaphotporn [44]	**67**	- Post/ant (PA) cervical mobilization - random mobilization (PA, right or left)	1.4 (SD 5.2) −0.4 (SD 7.6)	1.8 (SD 5.4) −0.4 (SD 5.9)	−0.2 (SD 4.0) 0.6 (SD 4.1)	0.9 (SD 4.2) 1.5 (SD 7.8)	1.2 (SD 5.9) 1.2 (SD 6.1)	2.7 (SD 5.3) 2.0 (SD 5.8)	No significant effect on ROM		Yes and no
Tuttle [41]	**67**	- PA cervical mobilization at symptomatic level - Placebo (PA mobilization but asymptomatic side)									
- Nothing (lying down)	* initial values flexion/extension ROM =119° (SD-17)	* initial values flexion/extension ROM=119° (SD-17)	* initial values lateral flexion ROM=93° (SD-12)	* initial values lateral flexion ROM=93° (SD-12)	* initial values rotation ROM=93° (SD-12)	* initial values rotation ROM=93° (SD-12)	No significant ROM increase	* No differences. pre/post treatment values are given but only shown in a graph.	Not tested		
Passmore [39]	**50**	- SMT(C1-C2) - Nothing (wait 5’)	No difference No difference	No difference No difference	No difference No difference	No difference No difference	3.75 No difference	-	SMT increases cervical active ROM		Not tested

**Table 5:** Effects of SMT on lumbar range of motion

**Author**	**Quality score /100**	**Treatment and control activity (sham, other or nothing)**	**Mean ROM measures in degrees pre-treatment (SD) Flexion**	**Mean ROM measures in degrees pre-treatment (SD) Extension**	**Mean ROM measures in degrees post-treatment (SD) Flexion**	**Mean ROM measures in degrees post-treatment (SD) Extension**	**Differences pre –post treatment in degrees Flexion**	**Differences pre –post treatment in degrees Extension**	**Comments**	**Were the differences in outcome tested between groups and were they significant?**
Konstantinou [46]	83	- Mobilization (n=26) - Lying down (n=26)	69.5 (19.0)	21.9 (10.2)	76.7 (22.4) 69.7 (21.5)	24.0 (11.0) 21.2 (11.1)	7.2 0.2	2.1 −0.7	Values pre-treatment are baseline for both groups. Small changes.	Yes and no
Stamospapastomous [47]	83	- SMT(lumbar) (n=32) - Mobilization (n=32)	54.22 (12.76) 54.11 (11.13)	27.02 (14.42) 22.8 (10.0)	56.07 (12.22) 54.81 (11.36)	28.56 (14.23) 23.66 (11.39)	1.85 0.7	1.54 0.86	No significant effect for the whole group, but some individual effects reported by author.	Not tested
Goodsell [45]	69	- PA mobilization - Nothing	A=105 (11) B=95 (22)	A=52 (8) B=46 (14)	A=106 (13) B=97(21) A=108 (12) B=95 (24)	A=52 (11) B=47 (14) A=52 (9) B=47 (14)	0.9 1.4	0.9 1.4	No effect on ROM.	Yes and no

**Table 6:** Effects of SMT on hip flexion range of motion

**Author**	**Quality score /100**	**Treatment and control activity (sham, other or nothing)**	**Pre-test ROM in degrees relative to vertical plane**	**Post-test ROM in degrees relative to vertical plane**	**Changes in %**	**Summarized results given by authors**	**Were the differences in outcome tested between groups and were they significant?**
Pollard [48]	33	- Sacroiliac manipulation (n=18) - Sham=mastoid pressure (n=16)	62.22 (2.40) 67.06 (3.23)	60.06 (2.22) 66.37 (3.21)	3.47 1.03	A single manipulation of the sacroiliac joint did not significantly affect hip ROM.	Yes and no

Outcomes were reported as either degrees or millimeters and there were generally no large improvements in ROM. In fact, although a statistically significant effect of SMT was seen in several studies, the differences between pre and post treatment, when clearly reported, were, for the best results, in the order of 3.5mm for mouth opening ( [34]) and 10° ( [37]) for unilateral cervical rotation.

A positive effect of SMT was reported in both of the studies where mouth opening was assessed after cervical manipulation (+3.5mm ( [34]) and 1.5mm ± 1.5 [35]). In five of the nine studies on cervical ROM ( [40], [37], [42], [43], and [39]) a positive effect was reported, whereas the remaining four studies ( [36], [41], [38], and [44]) did not show improvement. See Table 4 for examples of increased ROM in the studies with significant results.

None of the three studies of the lumbar spine (Table 5) showed an effect of SMT on lumbar ROMs ( [46], [47], and [45]) and one study ( [48]) of sacroiliac manipulation (Table 6) reported no effect on the ROM of the hip joint.

The lack of homogeneity in the various reports made a meta-analysis impractical. For example, different areas of the spine were studied, all of which have different movement patterns. Concerning the cervical region, study [42] measured only rotation and not all three planes; study [40] showed only the baseline values and not the post-manipulative values. Study [41] did not report exact measures but showed the results in a graph. Finally, study [39] reported only exact numerical values for the significant findings but provided no estimates for those with non-significant differences. Also the study populations differed between papers, some including patients with back problems and others using healthy people. Only two studies, ( [41] and [35]), compared the outcome of SMT in three groups: treatment, sham procedure and a control group which did not receive any type of treatment. The first of these two studies, which examined cervical ROM, did not find an effect, whereas the second, which examined mouth opening, did.

Nine studies compared the action of SMT to only a sham procedure ( [36], [48], [40], [37], [43], [34], [38], [44], and [47]). Five of them ( [40], [37], [43], [34], and [47]) found the outcome of SMT to be statistically significant but four ( [36], [48], [38], and [44]) did not.

Four authors compared the action of SMT to the outcome in a control group which did not receive any treatment ( [45], [46], [42], and [39]). The first of these studies ( [45]) did not find any difference between the outcomes after SMT and the control process, whereas the three others did ( [46], [42], and [39]).

Finally, if articles are listed by total quality score, the seven highest ranked studies ( [35], [43], [46], [40], [47], [42], and [37]), with scores ranging from 92 to 77, showed significant positive effects of SMT on ROM. Continuing down the list from scores of 69 to 33, studies [45], [38], [36], [41], [44], and [48] reported no significant differences in the outcomes between groups, while studies [34] and [39] did report significant effects of SMT.

## Discussion

### Summary of results

This appears to be the first systematic critical literature review of the immediate effect of SMT on ROM. The fifteen articles that met our inclusion criteria for review did not provide a coherent picture; some studies found a small effect, others none. The differences in results between studies were related to the quality of studies, with the better studies reporting significant findings. Interestingly, it did not matter if SMT was compared with a sham treatment or if it was compared with some type of control procedure, suggesting that results were unaffected by expectation or observation bias. The only studies showing a positive effect in the spine were those performed in the cervical spine, while no effect was discerned after pelvic or lumbar treatment. When positive findings were present, they were generally modest.

### Heterogeneous methods make meta-analysis unsuitable

Many different tools are used in research and in clinical practice to measure ROM: single/double/triple inclinometers, goniometers, a rangiometer, tape measures, visual estimation, spine motion analyzer, etc. It was not the purpose of this review to describe and compare these methods, but we refer to the literature concerning their reliability and validity [33,49-52]. The main point is that there are quite a number of studies on the topic but that the measurement tools used often differed from one study to another, making it difficult to compare results between studies. In addition, this multitude of outcome variables plus other differences such as choice of study population and area of manipulation make it impossible to combine results in a meaningful meta-analysis.

### Why such small effects- if any?

The small improvement in ROM, when an improvement was seen at all, may come as a surprise to those who have clinical experience with SMT. However, perhaps in clinical practice, the biomechanical effect of SMT is influenced by a reduction in pain. In this review, we did not take into account changes in level of pain, but rather focused on ROM, which is a less common outcome variable in randomized clinical trials on SMT and back pain [11,46]. The choice of researchers to avoid measures of ROMs as outcome variables might well be because SMT does not generally produce substantial changes in ROMs.

Another possibility for this discrepancy between the clinical experience and the results of this review is that ROMs may improve gradually as treatment progresses over days or weeks. Our review dealt only with the immediate effects of SMT, in order not to confuse the effects of SMT with those of other factors that could come into play over a period of time. Other possible explanations are that SMT might produce changes in kinematics (i.e. how the spine moves) rather than changes in the total ROM. It is also possible that changes in one manipulated joint among several in a spinal region would not contribute greatly to changes in regional movement.

### Significant effects only in the cervical spine

In this review, SMT only showed discernible effects in the cervical spine. There may be several explanations for this. First, gross ranges of motion are largest in the cervical spine and so there is the potential for treatment in that area to cause a larger, hence measureable, effect. Secondly, more than 50% of the total rotation of the cervical spine is attributed to movement at a single level, the atlanto-axial joint [53]. Hence, SMT targeting the atlanto-axial joint could potentially lead to a substantial overall increase in the rotation of the entire cervical spine. In the lumbar and thoracic spine no single segmental level is responsible for a substantial proportion of movement of the entire region. Therefore, an increase in movement at a single level below the neck is less likely to result in a large change in regional ROM, as shown in this review.

### Post hoc analysis

In order to investigate this matter further, all articles that tested the effect of SMT specifically in the upper cervical spine were re-analyzed, in relation to the size of the effect. Two studies [39,40] treated C1-C2, with different results: the first one [39], showed a difference pre/post treatment (3.75 degrees) only for right neck rotation. The other [40], showed that SMT significantly increased neck flexion and rotation (see Table 4). Studies [37,41] treated at the C3-C6 level and they also had different results: [37] showed that SMT was more effective than control mobilization, whereas [41] showed no significant effect on ROM. One study [42] was conducted at the thoracic level and showed improved cervical ROM. Finally four studies [36,38,43,44] treated a clinically determined area and only one [36] showed improved cervical ROM with SMT. These results, therefore, do not corroborate the theory that upper cervical SMT has a particularly obvious effect on cervical rotation. Further study is obviously needed on this aspect of spinal ROM.

### Technical challenges in measuring ROM

There are also some technical issues that have to be taken into account in interpreting the results presented herein. For example, measurements of ROM face the problem of where to start and stop; i.e. how to identify the neutral or starting position. Therefore, measurements are probably more accurate when the full ROM is reported. Although most studies in our review did measure the full range, for some reason they reported on each individual movement component (e.g. left rotation and right rotation).

It is also possible that ROM does increase immediately following treatment but that the methods used in the reviewed studies did not capture this effect. In this regard, most of authors either claimed to be using a previously tested and acceptable instrument in relation to reliability, or they presented their own reliability study with acceptable results.

Interestingly, many studies of cervical ROM seem to concentrate on the reliability of measurement tools, but construct validity and what has been referred to as ‘responsiveness’ - the ability to capture change - are studied less often [54]. Whether a particular device is capable of accurately measuring ROM with appropriate precision is of utmost importance. However, it has been pointed out [33] that certain methodological procedures, rather than the device itself, are problematic, at least for cervical ROMs. Such items are wide variations in study designs, the characteristics of the observers and study populations, whether warm-ups were allowed, whether the movements were active or passive, and the time intervals between repeated measurements. Furthermore, it is difficult to assess ROMs, and outcomes may not be reliable if there is pain in the examined area [55]. These were all items that we examined in our critical review of articles, and it was apparent that a number of studies did not take these concerns into consideration.

### Methodological considerations of this review

As with all systematic literature reviews, it is likely that not all relevant articles were found, and the review process itself has a subjective element even with the systematic approach of using checklists. Other researchers may prefer to scrutinize other methodological items and may judge the contents differently. However, as there were no disagreements between the two primary reviewers, the protocol appears to have been appropriate and clearly defined.

Research findings are of course dependent on the study methodology; it is often seen that the weakest studies have the “best” results. However, in this review, the methodological score was, generally, associated with study outcome in the opposite way, i.e. studies with better quality scores were more likely to show statistically significant differences, suggesting that the effects of SMT on ROM are robust, albeit not very large. Nevertheless, the fact that there was no set cut-point for methodological acceptability and because the evidence tables report on both descriptive and quality items, the reader is able to make his/her own additional analysis of the data, should this be relevant.

### Implications of findings for future research

It has been shown that it is difficult to evaluate ROM if there is pain [55]. On the other hand, it does not seem relevant to manipulate non-painful articulations in order to study the effect on movement, as these likely already have a full ROM. Therefore, it is difficult to know whether clinical improvements following SMT in patients with neck pain [11] or patients with low-back pain [1] are due mainly to improved biomechanical status, a direct reduction of pain, or a combination of the two. A previous review concluded that there is a direct action of SMT on experimentally induced pain [26] but the present review cannot isolate a general direct effect of SMT on ROM. Consequently, other information seems to be necessary to better understand the relationship between pain and motion in relation to SMT.

#### Implications of findings for clinical practice

The results do not support the concept that SMT has an immediate, strong and obvious effect on ROM in the human spine. In clinical practice, it is possible that measurements of ROM should be clearly separated from the experience of pain in relation to movements.

## Conclusion

Based on the studies reviewed herein, SMT sometimes seems to have a small effect on ROM, at least in the cervical spine. The studies reviewed did not reveal an effect of SMT on temporomandibular, lumbar or hip ROMs. Future studies into ROM should take into account various stages of degeneration, should standardize the experimental situation, and should include both pain and ROM as outcome variables. It would also be useful to include study subjects who actually have a decreased ROM, in order to have the potential for improvement. Also, it seems obvious that the measured movement should have the potential to improve with the treatment. Therefore, the effect of SMT on the upper cervical spine should be tested in relation to rotation and treatment to the lower cervical spine in relation to lateral bending, flexion and extension. The full ROM should be measured with validated instruments and all estimates should be clearly reported, including full statistical analysis.

## Competing interests

Authors declare there are no conflicts of interest.

## Authors’ contributions

All authors instigated this review. MM and CLY designed the check-lists. MM and BB searched in the databases. MM and CLY reviewed the literature and wrote the first draft. BB, MDC and MAA provided expertise on the topic, assisted with the literature review and provided critical comments to the first draft. All authors reviewed the final manuscript and approved the final version.

## References

[B1] Rubinstein S Van Middelkoop M Assendelft W De Boer M Van Tulder M Spinal manipulative therapy for chronic low-back pain: an update of a cochrane review Spine 2011 36 13 E825 E846 10.1097/BRS.0b013e3182197fe1 21593658

[B2] Leach RA** *The chiropractic theories* **2004 Fourth Lippincott Williams & Wilkins, Baltimore 131–197 235–246

[B3] Walter H The biomechanics of spinal manipulation J Bodyw Mov Ther 2010 14 3 280 286 10.1016/j.jbmt.2010.03.004 20538226

[B4] Maigne J-Y Vautravers P Mechanism of action of spinal manipulative therapy Joint Bone Spine 2003 70 5 336 341 10.1016/S1297-319X(03)00074-5 14563460

[B5] Vernon H Mrozek J A revised definition of manipulation J Manipulative Physiol Ther 2005 28 1 68 72 10.1016/j.jmpt.2004.12.009 15726038

[B6] Ianuzzi A Khalsa P Comparison of human lumbar facet joint capsule strains during simulated high-velocity, low-amplitude spinal manipulation versus physiological motions Spine J 2005 5 277 290 10.1016/j.spinee.2004.11.006 15863084 PMC1315283

[B7] Herzog W The biomechanics of spinal manipulation J Bodyw Mov Ther 2010 14 3 280 286 10.1016/j.jbmt.2010.03.004 20538226

[B8] Sung P Kang Y-M Pickar J Effect of spinal manipulation duration on low threshold mechanoreceptors in lumbar paraspinal muscles: a preliminary report Spine 2005 30 1 115 122 15626991 10.1097/01.brs.0000147800.88242.48

[B9] Stoddard A Osteopathic techniques of manipulation Physiotherapy 1970 56 1 29 30 5467143

[B10] Snodgrass SJ Rivett DA Robertson VJ Stojanovski E Forces applied to the cervical spine during posteroanterior mobilization J Manipulative Physiol Ther 2009 32 1 72 83 10.1016/j.jmpt.2008.09.012 19121465

[B11] Gross A Miller J D’Sylva J Burnie SJ Goldsmith CH Graham N Haines T Brønfort G Hoving JL Manipulation or mobilisation for neck pain: a Cochrane review Man Ther 2010 15 4 315 333 10.1016/j.math.2010.04.002 20510644

[B12] Forand D Drover J Suleman Z Symons B Herzog W The forces applied by female and male chiropractors during thoracic spinal manipulation J Manipulative Physiol Ther 2004 27 1 49 56 10.1016/j.jmpt.2003.11.006 14739874

[B13] Dishman JD Burke J Spinal reflex excitability changes after cervical and lumbar spinal manipulation: a comparative study Spine J 2003 3 3 204 212 10.1016/S1529-9430(02)00587-9 14589201

[B14] Roy RA Boucher JP Comtois AS Paraspinal cutaneous temperature modification after spinal manipulation at L5 J Manipulative Physiol Ther 2010 33 4 308 314 10.1016/j.jmpt.2010.03.001 20534318

[B15] Bolton PS Budgell B Visceral responses to spinal manipulation J Electromyogr Kinesiol http://www.sciencedirect.com/science/article/pii/S1050641112000478 10.1016/j.jelekin.2012.02.016 22440554

[B16] DeVocht JW Pickar JG Wilder DG Spinal manipulation alters electromyographic activity of paraspinal muscles: a descriptive study J Manipulative Physiol Ther 2005 28 7 465 471 10.1016/j.jmpt.2005.07.002 16182019

[B17] Lehman G Kinesiological research: the use of surface electromyography for assessing the effects of spinal manipulation J Electromyogr Kinesiol 0 http://www.sciencedirect.com/science/article/pii/S1050641112000417 10.1016/j.jelekin.2012.02.010 22425147

[B18] Pickar JG Neurophysiological effects of spinal manipulation Spine J 2002 2 5 357 371 10.1016/S1529-9430(02)00400-X 14589467

[B19] Botelho MB Andrade BB Effect of cervical spine manipulative therapy on judo athletes' grip strength J Manipulative Physiol Ther 2012 35 1 38 44 10.1016/j.jmpt.2011.09.005 22079053

[B20] Grindstaff TL Hertel J Beazell JR Magrum EM Ingersoll CD Effects of lumbopelvic joint manipulation on quadriceps activation and strength in healthy individuals Man Ther 2009 14 4 415 420 10.1016/j.math.2008.06.005 18805726

[B21] Taylor HH Murphy B Altered sensorimotor integration with cervical spine manipulation J Manipulative Physiol Ther 2008 31 2 115 126 10.1016/j.jmpt.2007.12.011 18328937

[B22] Bialosky JE Bishop MD Robinson ME Zeppieri G Jr George SZ Spinal manipulative therapy has an immediate effect on thermal pain sensitivity in people with low back pain: a randomized controlled trial Phys Ther 2009 89 12 1292 1303 10.2522/ptj.20090058 19797305 PMC2794479

[B23] Fernández-Carnero J Fernández-de-las-Peñas C Cleland J Immediate hypoalgesic and motor effects after a single cervical spine manipulation in subjects with lateral epicondylalgia J Manipulative Physiol Ther 2008 31 9 675 681 10.1016/j.jmpt.2008.10.005 19028251

[B24] Ruiz-Sáez M Fernández-de-las-Peñas C Blanco C Martínez-Segura R García-León R Changes in pressure pain sensitivity in latent myofascial trigger points in the upper trapezius muscle after a cervical spine manipulation in pain-free subjects J Manipulative Physiol Ther 2007 30 8 578 583 10.1016/j.jmpt.2007.07.014 17996549

[B25] Sterling M Jull G Wright A Cervical mobilisation: concurrent effects on pain, sympathetic nervous system activity and motor activity Man Ther 2001 6 2 72 81 10.1054/math.2000.0378 11414776

[B26] Coronado RA Gay CW Bialosky JE Carnaby GD Bishop MD George SZ Changes in pain sensitivity following spinal manipulation: A systematic review and meta-analysis J Electromyogr Kinesiol 2012 http://www.sciencedirect.com/science/article/pii/S1050641112000065 10.1016/j.jelekin.2011.12.013 PMC3349049 22296867

[B27] Millan M Leboeuf-Yde C Budgell B Amorim M-A The effect of spinal manipulative therapy on experimentally induced pain: a systematic literature review Accepted for publication in Chiropractic Manual Therapies 10.1186/2045-709X-20-26 PMC3527169 22883534

[B28] Liberati A Altman D Tetzlaff J Mulrow C Gøtzsche P Ioannidis J Clarke M Devereaux P Kleijnen J Moher D The PRISMA statement for reporting systematic reviews and meta-analyses of studies that evaluate health care interventions: explanation and elaboration J Clin Epidemiol 2009 62 10 e1 e34 10.1016/j.jclinepi.2009.06.006 19631507

[B29] Moher D Hopewell S Schulz K Montori V Gøtzsche P Devereaux P Elbourne D Egger M Altman D CONSORT 2010 explanation and elaboration: updated guidelines for reporting parallel group randomised trials Int J Surg 2011 1 27 54

[B30] Furlan A Pennick V Bombardier C Van Tulder M 2009 updated method guidelines for systematic reviews in the Cochrane back review group Spine 2009 34 18 1929 1941 10.1097/BRS.0b013e3181b1c99f 19680101

[B31] Colloca CJ Keller TS Moore RJ Gunzburg R Harrison DE Effects of disc degeneration on neurophysiological responses during dorsoventral mechanical excitation of the ovine lumbar spine J Electromyogr Kinesiol 2008 18 5 829 837 10.1016/j.jelekin.2007.02.017 17468010

[B32] Colloca C Intervertebral disc degeneration reduces vertebral motion responses Spine 2007 32 19 E544 E550 10.1097/BRS.0b013e318145ac39 17762796

[B33] Jordan K Assessment of published reliability studies for cervical spine range-of-motion measurement tools J Manipulative Physiol Ther 2000 23 3 180 195 10.1016/S0161-4754(00)90248-3 10771503

[B34] Mansilla-Ferragut P Fernández-de-las Peñas C Alburquerque-Sendín F Cleland JA Boscá-Gandía JJ Immediate effects of atlanto-occipital joint manipulation on active mouth opening and pressure pain sensitivity in women with mechanical neck pain J Manipulative Physiol Ther 2009 32 2 101 106 10.1016/j.jmpt.2008.12.003 19243721

[B35] Oliveira-Campelo N Rubens-Rebelatto J Marti N Vallejo F Alburquerque-Sendi N Fernandez-de-Las-Penas C The immediate effects of atlanto-occipital joint manipulation and suboccipital muscle inhibition technique on active mouth opening and pressure pain sensitivity over latent myofascial trigger points in the masticatory muscles J Orthop Sports Phys Ther 2010 40 5 310 317 20436241 10.2519/jospt.2010.3257

[B36] Cassidy JD The immediate effect of manipulation versus mobilization on pain and range of motion in the cervical spine: a randomized controlled trial J Manipulative Physiol Ther 1992 15 9 570 575 1469341

[B37] Martínez-Segura R Fernández-de-las-Peñas C Ruiz-Sáez M López-Jiménez C Rodríguez-Blanco C Immediate effects on neck pain and active range of motion after a single cervical high-velocity low-amplitude manipulation in subjects presenting with mechanical neck pain: a randomized controlled trial J Manipulative Physiol Ther 2006 29 7 511 517 10.1016/j.jmpt.2006.06.022 16949939

[B38] McClatchie L Laprade J Martin S Jaglal SB Richardson D Agur A Mobilizations of the asymptomatic cervical spine can reduce signs of shoulder dysfunction in adults Man Ther 2009 14 4 369 374 10.1016/j.math.2008.05.006 18752983

[B39] Passmore SR Burke JR Good C Lyons JL Dunn AS Spinal manipulation impacts cervical spine movement and Fitts' task performance: a single-blind randomized before-after trial J Manipulative Physiol Ther 2010 33 3 189 192 10.1016/j.jmpt.2010.01.007 20350671

[B40] Whittingham W Nilsson N Active range of motion in the cervical spine increases after spinal manipulation (toggle recoil) J Manipulative Physiol Ther 2001 24 9 552 555 10.1067/mmt.2001.118979 11753327

[B41] Tuttle N Barrett R Laakso L Relation between changes in posteroanterior stiffness and active range of movement of the cervical spine following manual therapy treatment Spine 2008 33 19 E673 E679 10.1097/BRS.0b013e31817f93f9 18758348

[B42] Krauss J Creighton D Ely JD Podlewska-Ely J The immediate effects of upper thoracic translatoric spinal manipulation on cervical pain and range of motion: a randomized clinical trial Journal of Manual & Manipulative Therapy (Journal of Manual & Manipulative Therapy) 2008 16 2 93 99 19119394 10.1179/106698108790818530 PMC2565124

[B43] Kanlayanaphotporn R Chiradejnant A Vachalathiti R The immediate effects of mobilization technique on pain and range of motion in patients presenting with unilateral neck pain: a randomized controlled trial Arch Phys Med Rehabil 2009 90 2 187 192 10.1016/j.apmr.2008.07.017 19236972

[B44] Kanlayanaphotporn R Chiradejnant A Vachalathiti R Immediate effects of the central posteroanterior mobilization technique on pain and range of motion in patients with mechanical neck pain Disabil Rehabil 2010 32 8 622 628 10.3109/09638280903204716 20205574

[B45] Goodsell M Lee M Latimer J Short-term effects of lumbar posteroanterior mobilization in individuals with low-back pain J Manipulative Physiol Ther 2000 23 5 332 342 10863253

[B46] Konstantinou K Foster N Rushton A Baxter D Wright C Breen A Flexion mobilizations with movement techniques: the immediate effects on range of movement and pain in subjects with low back pain J Manipulative Physiol Ther 2007 30 3 178 185 10.1016/j.jmpt.2007.01.015 17416271

[B47] Stamos-Papastamos N Petty NJ Williams JM Changes in bending stiffness and lumbar spine range of movement following lumbar mobilization and manipulation J Manipulative Physiol Ther 2011 34 1 46 53 10.1016/j.jmpt.2010.11.006 21237407

[B48] Pollard H Ward G The effect of sacroiliac manipulation on hip flexion range of motion Australasian Chiropractic & Osteopathy 1997 6 3 80 84 17987153 PMC2050632

[B49] Prushansky T Deryi O Jabarreen B Reproducibility and validity of digital inclinometry for measuring cervical range of motion in normal subjects Physiother Res Int 2010 15 1 42 48 19554615 10.1002/pri.443

[B50] O’Sullivan K O’Sullivan L Campbell A O’Sullivan P Dankaerts W Towards monitoring lumbo-pelvic posture in real-life situations: Concurrent validity of a novel posture monitor and a traditional laboratory-based motion analysis system Man Ther 2012 17 1 77 83 10.1016/j.math.2011.09.006 22015373

[B51] Gelalis I DeFrate L Stafilas K Pakos E Kang J Gilbertson L Three-dimensional analysis of cervical spine motion: reliability of a computer assisted magnetic tracking device compared to inclinometer Eur Spine J 2009 18 2 276 281 10.1007/s00586-008-0853-0 19096884 PMC2899335

[B52] Assink N Bergman GJD Knoester B Winters JC Dijkstra PU Assessment of the cervical range of motion over time, differences between results of the Flock of Birds and the EDI-320: A comparison between an electromagnetic tracking system and an electronic inclinometer Man Ther 2008 13 5 450 455 10.1016/j.math.2007.05.012 17681865

[B53] Bogduk N Mercer S Biomechanics of the cervical spine. I: Normal kinematics Clin Biomech 2000 15 633 648 10.1016/S0268-0033(00)00034-6 10946096

[B54] de Koning C van den Heuvel S Staal J Smits-Engelsman B Hendriks E Clinimetric evaluation of active range of motion measures in patients with non-specific neck pain: a systematic review Eur Spine J 2008 17 7 905 921 10.1007/s00586-008-0656-3 18427843 PMC2443270

[B55] Lehman GJ Biomechanical assessments of lumbar spinal function. How low back pain sufferers differ from normals. Implications for outcome measures research. Part I: kinematic assessments of lumbar function J Manipulative Physiol Ther 2004 27 1 57 62 10.1016/j.jmpt.2003.11.007 14739876

[B56] Preferred Reporting Items for Systematic Reviews and Meta-Analyses: The PRISMA Statementwww.prisma-statement.org 10.7326/0003-4819-151-4-200908180-00135 19622511

